# *Eimeria tenella* protein trafficking: differential regulation of secretion *versus* surface tethering during the life cycle

**DOI:** 10.1038/s41598-017-04049-1

**Published:** 2017-07-04

**Authors:** V. Marugan-Hernandez, E. Long, D. Blake, C. Crouch, F. Tomley

**Affiliations:** 1 The Royal Veterinary College, University of London, Hawkshead Lane, North Mymms, AL9 7TA UK; 2 MSD Animal Health, Walton Manor, Milton Keynes MK7 7AJ UK

## Abstract

*Eimeria* spp. are intracellular parasites that have a major impact on poultry. Effective live vaccines are available and the development of reverse genetic technologies has raised the prospect of using *Eimeria* spp. as recombinant vectors to express additional immunoprotective antigens. To study the ability of *Eimeria* to secrete foreign antigens or display them on the surface of the sporozoite, transiently transfected populations of *E. tenella* expressing the fluorescent protein mCherry, linked to endogenous signal peptide (SP) and glycophosphatidylinositol-anchor (GPI) sequences, were examined. The SP from microneme protein EtMIC2 (SP2) allowed efficient trafficking of mCherry to cytoplasmic vesicles and following the C-terminal addition of a GPI-anchor (from surface antigen EtSAG1) mCherry was expressed on the sporozoite surface. In stable transgenic populations, mCherry fused to SP2 was secreted into the sporocyst cavity of the oocysts and after excystation, secretion was detected in culture supernatants but not into the parasitophorous vacuole after invasion. When the GPI was incorporated, mCherry was observed on the sporozites surface and in the supernatant of invading sporozoites. The proven secretion and surface exposure of mCherry suggests that antigen fusions with SP2 and GPI of EtSAG1 may be promising candidates to examine induction of protective immunity against heterologous pathogens.

## Introduction

Apicomplexan parasites can cause many human and/or animal diseases. This group of intracellular protozoa includes malarial parasites of the genus *Plasmodium* and zoonotic pathogens such as *Cryptosporidium* species and *Toxoplasma gondii*. Many apicomplexans have a persistent impact on animal welfare and livestock production including species of *Babesia*, *Cystoisospora*, *Eimeria*, *Neospora* and *Theileria*. Apicomplexan biology varies between genera but includes complex developmental life cycles and obligate interactions with host cells. They are difficult parasites to control and few vaccines or completely effective drug therapies are available.

*Eimeria* species can cause coccidiosis, an intestinal infection that results in malabsorption, diarrhoea and/or haemorrhage with an enormous impact on livestock, particularly poultry. Coccidiosis is controlled mainly through prophylactic use of anticoccidial drugs. Live and live-attenuated *Eimeria* vaccines are available but their usage is currently restricted to a small proportion of the global poultry industry because of limitations in production and the relatively high cost compared to anticoccidial drugs^1, 2^. The use of live *Eimeria* vaccines as vectors to express immunoprotective antigens from additional poultry pathogens could potentially add significant value by rendering vaccines multivalent and able to protect chickens against a range of different diseases. A number of studies have tested the ability of *Eimeria tenella* to express heterologous antigens with variable results based on the detection of specific antibodies^3, 4^ or the development of immune protection against challenge^5^. Considerable work is needed to optimise antigen delivery and better characterise immune responses induced by transgenic parasites against the foreign antigens. Pathogen-derived antigens have been expressed using *Eimeria* under the control of different promoter regions, but always with a cytosolic site of expression. Studies in the closely related parasite *T. gondii* suggest that the subcellular localization of antigens within the parasite can lead to variation in the nature of the immune response induced. Secreted antigens are much more efficient in stimulating and activating both CD8^+ ^^6^ and CD4^+ ^^7^ T-cells, compared to intracellular antigens (expressed in the cytosol or in non-secreting organelles). For transgenic *E. tenella* expressing enhanced yellow fluorescent protein (EYFP), both compartmentalized and cytosolic EYFP induced antigen-specific cell proliferation, however, the former showed higher levels of antigen specific lymphocyte proliferation^3^, indicating that the location of the expressed antigen may be crucial for *Eimeria* to function as an effective vaccine vector.

Apicomplexan parasites possess a complex endomembrane system with specialised secretory organelles (micronemes, rhoptries and dense granules) that are important in parasite invasion and interaction with the host cell^8^. The signals and pathways followed by proteins to reach their final destinations are not yet completely defined^9^. Most knowledge has been assembled in *T. gondii* where an endosomal-like system of protein trafficking has been described^10^ and specialised N-terminal pro-peptide targeting signals identified in several proteins destined for storage in the rhoptry or microneme organelles before their release during invasion. These include the propeptides of *T. gondii* rhoptry protein (ROP) TgROP1 (which has at least two rhoptry targeting signals^11, 12^), subtilisin TgSUB1^13^ and the microneme proteins (MIC) TgM2AP^14^, TgMIC3^15^ and TgMIC5^16^. It has also been shown that the propeptide of the *E*. *tenella* microneme protein EtMIC5 has an interchangeable function when introduced into *T. gondii* ^17^. The trafficking of transmembrane rhoptry and microneme proteins including TgMIC2 and TgROP2 also depends on the presence of tyrosine residues in their cytoplasmic tails^18, 19^, suggesting conservation of tyrosine-dependant sorting found in higher eukaryotes^20^. In *Toxoplasma*, some GTPases such as the dynamin DrpB^21^, or the Rab5A and Rab5C^22^ and sortilin-like receptors (TgSORTLR)^23^, have a critical role in microneme and rhoptry biogenesis as well as in the transport of their contents, following at least two different pathways trafficking to the micronemes. In contrast no sorting signals have been defined for proteins destined for dense granules and these appear to be storage organelles within the constitutive or ‘default’ secretory pathway, housing proteins that are discharged into the newly formed parasitophorous vacuole (PV) following parasite invasion of the host cell^24^. Recently, an additional pathway has been proposed for a subset of dense granule proteins that are targeted beyond the vacuole to the host cell nucleus^25^. The trafficking pathway followed by glycophosphatidylinositol (GPI)-linked surface antigens (SAG) has not been fully elucidated, although GPI-anchors at the C-terminal of these proteins appear to contain all of the targeting signals required for this external localization^24^.

Little is known about protein targeting and trafficking specifically in *Eimeria* spp. due in part to less advanced development of reverse genetic tools compared to those available for *T. gondii*. The use of *in vitro* models is crucial for the study of protein localization and, although *E. tenella* cannot complete its entire endogenous development *in vitro* (sporogony, several rounds of schizogony, gametogony and zygote formation leading to the formation of new oocysts), we have reliable methods for *in vitro* release of sporozoites from sporulated oocysts, and the infection of cell monolayers with purified sporozoites that result in completion of one single round of schizogony and the release of first generation merozoites. In the present study populations of transgenic parasites were produced that express the mCherry fluorescent reporter fused at its N-terminus to *Eimeria* microneme protein signal peptides, with or without the addition of a C-terminal glycophosphatidylinositol anchor signal sequence derived from an *Eimeria* surface protein. The potential secretion or surface attachment of expressed mCherry in these novel transgenic populations was followed as parasites progressed through sporulation and subsequently in sporozoites, schizonts and first generation merozoites (in cell culture).

## Results

### The signal peptide of EtMIC2 (SP2) can translocate mCherry into the endomembrane network in transiently transfected sporozoites

The presence of a SP is necessary for proteins to translocate from their ribosomal site of translation into the lumen of the endoplasmic reticulum (ER) and from there traffic in the endomembrane system to their final location within the cell or beyond. Without a SP the mCherry protein is expected to remain in the parasite cytosol; the presence of a SP without any additional targeting sequence should result in the protein being trafficked as soluble cargo through the parasite secretory pathway with its eventual release from the endomembrane system at the parasite surface. To evaluate this in sporozoites of *E. tenella*, we transfected sporozoites with plasmids where mCherry was fused to three different SP: SP2, SP5 or SP9 corresponding to microneme proteins EtMIC2, EtMIC5 or EtMIC9, respectively; under the control of genomic sequences corresponding to 5′*EtMIC2*, 5′*EtMIC5*, 5′*EtMIC9* or 5′*EtTIF* (a genomic region upstream of gene ETH_00025365 encoding a putative translation initiation factor^4^) (Table 1). These upstream genomic regions were also analysed without the SP (Table 1). The different plasmids also contained a *mCitrine* cassette expressed as a soluble cytosolic protein under the control of 5′*EtMIC1* used to detect and select transgenic parasites by Flow-activated cell sorting (FACS). Transfected sporozoites were added to fresh Madin-Darby bovine kidney (MDBK) cell monolayers and fluorescence was observed 24 hours later.

**Table 1:** Summary of fluorescence profiles of the sporozoites transfected with different plasmid constructs.

Plasmid ID	mCitrine Expression	mCherry Expression	mCherry Location	mCherry Pattern	mCherry Intensity
p5M2-mChe	+	+	cytosolic	Fig. 1a panel 1	Fig. 1b (↑)
p5M5-mChe	+	+	cytosolic	Fig. 1a panel 1	Fig. 1b (↑)
p5M9-mChe	+	−	n/a	Fig. 1a panel 3	Fig. 1b (0)
p5M2-SP2-mChe	+	+	anterior end	Fig. 1a panel 2	Fig. 1b (↑)
p5M5-SP5-mChe	+	−	n/a	Fig. 1a panel 3	Fig. 1b (0)
p5M9-SP9-mChe	+	−	n/a	Fig. 1a panel 3	Fig. 1b (0)
p5TIF-SP2-mChe	+	+	anterior end	Fig. 1a panel 2	Fig. 1b (↓)
p5TIF-SP5-mChe	+	+	cytosolic	n/a	Fig. 1b (↓)
p5TIF-SP9-mChe	+	+	cytosolic	n/a	Fig. 1b (↓)
p5TIF-mChe	+	+	cytosolic	Fig. 1a panel 1	Fig. 1b (↓)
pREPORTER (mChe)	−	+	cytosolic	Fig. 1a panel 1	Fig. 1b (↑)

**Figure 1:**
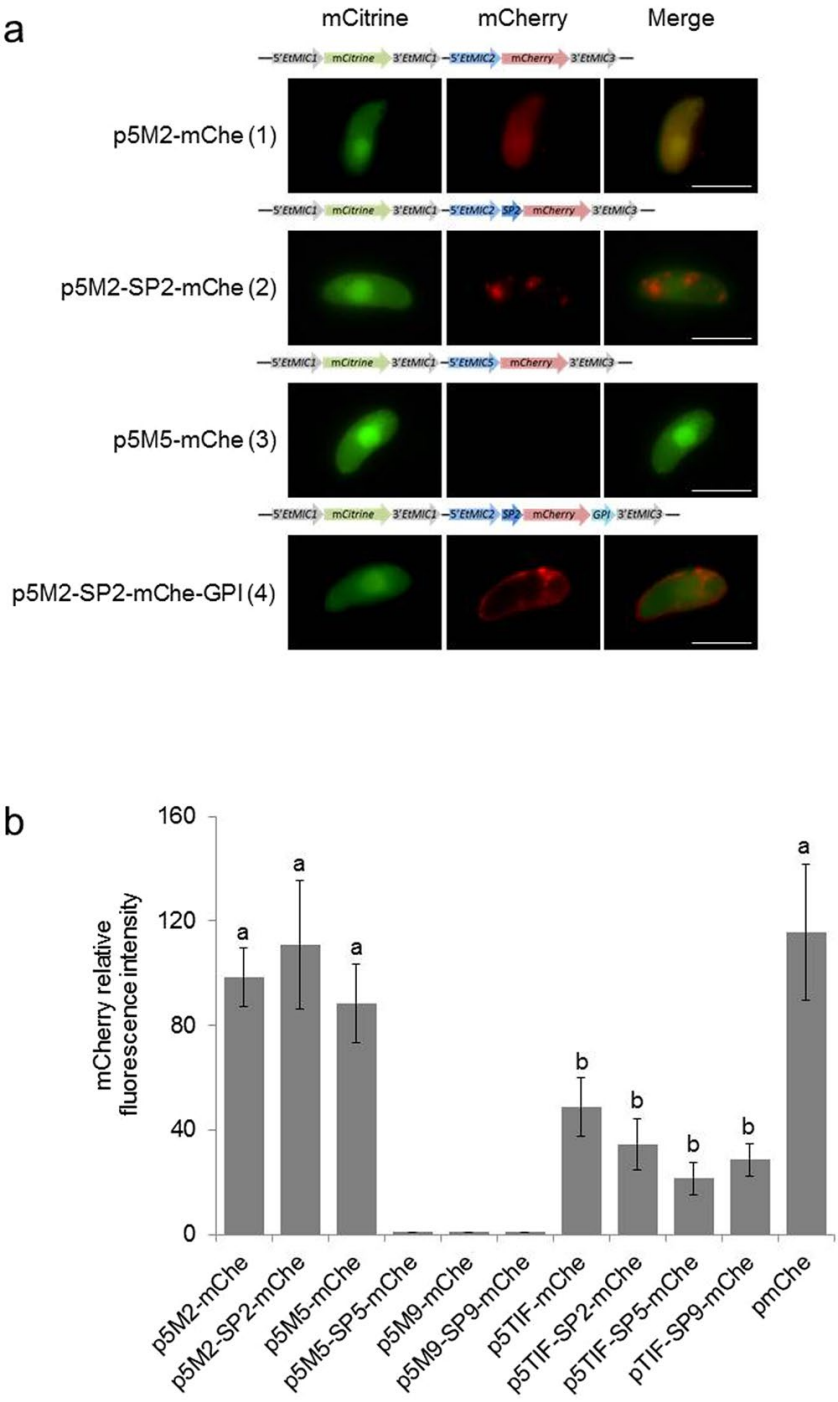
Study of *E. tenella* sporozoites transiently transfected with different plasmids and incubated with MDBK cells for 24 h (41 °C, 5% CO_2_). (**a**) Fluorescent patterns observed after transfection with: (1) p5M2-mChe plasmid (Table 2), mCherry was observed in the cytosol; (2) p5M2-SP2-mChe plasmid (Table 2), mCherry was observed as vesicles in the cytoplasm; (3) p5M5-mChe plasmid (Table 2), mCherry not observed; (4) p5M2-SP2-mChe-GPI plasmid (Table 2), mCherry was observed on the sporozoite surface. mCitrine was always observed in the cytosol of the transfected sporozoites. The plasmids constructs for each transfection are displayed above each panel. Bars represent 5 *µ*m. (**b**) Relative fluorescence intensity – values between 0 (pure black) and 255 (pure white) – of mCherry analysed by Image J of sporozoites transfected with the different plasmids; **a** and **b** indicate different significances (p < 0.05; one-way ANOVA with post hoc Bonferroni test).

Genomic sequences of 5′*EtMIC2* and 5′*EtMIC5*, were found to support the expression of mCherry resulting in strong red fluorescence in the cytoplasm (Fig. 1a panel 1; Fig. 1b; Table 1) when cloned upstream of the *mCherry* coding sequence without any SP. Intriguingly, when the corresponding SP sequences were introduced 5′*EtMIC2-sp2* (Fig. 1a panel 2; Table 1), but not 5′*EtMIC5-sp5* (Fig. 1a panel 3; Table 1), was found to support expression of the reporter and in this case the fused SP2-mCherry was visualised as vesicles in the parasite cytoplasm (Fig. 1a panel 2). This suggests that SP2 is able to translocate mCherry into the endomembrane network. The promoter region 5′*EtMIC9* was not functional either with or without its corresponding SP (Table 1) suggesting that the 1 kb region selected from upstream of the *EtMIC9* coding sequence lacked appropriate recognition sequences for initiation of transcription.

In another set of plasmids, genomic sequences corresponding to microneme-gene upstream regions were replaced by the constitutive promoter region 5′*EtTIF*. After transfection, the cytosolic red fluorescence observed (without any SP) was significantly less intense than when *mCherry* was linked to 5′*EtMIC2* or 5′*EtMIC5* sequences (Fig. 1b; Table 1) (p < 0.05, one-way ANOVA with post hoc Bonferroni test). The fused SP2-mCherry seemed to be restricted again to the anterior part of the sporozoite but at a lower intensity than when linked to 5′*EtMIC2* (Table 1; Fig. 1b). Likewise, both fused SP5-mCherry and SP9-mCherry could be detected faintly (Fig. 1b) in transfected sporozoites, however, the intensity was not enough to assign a specific subcellular localization.

Curiously, mCitrine (linked to the 5′*EtMIC1* promoter) was significantly less intense in sporozoites transfected with p5M2-mChe and p5M2-SP2-mChe (containing 5′*EtMIC2*) compared with sporozoites transfected with plasmids containing 5′*EtTIF* (p < 0.05, one-way ANOVA with post hoc Bonferroni test), which could be explained by the simultaneous expression of two promoters (5′*EtMIC1* and 5′*EtMIC2*) under a similar time regulation^26^.

### The addition of a GPI anchor to the fused SP2-mCherry targets the protein to the parasite surface in transiently transfected sporozoites

As described above, SP2 was able to translocate mCherry into the endomembrane system and the 5′*EtMIC2* promoter region conferred enough intensity to study SP2-mCherry directly by microscopy. Thus, plasmid p5M2-SP2-mChe (Table 2; Supplementary Fig. S1) was selected for C-terminal fusion with the GPI-anchor sequence of *E. tenella* surface antigen 1 (EtSAG1) (Table 2; Supplementary Fig. S1). In sporozoites transfected with p5M2-SP2-mChe-GPI, the mCherry protein was found to be expressed on the parasite surface (Fig. 1a panel 4).

**Table 2:** List of plasmids used in the study and their principal features.

Plasmid ID	1^st^ CASSETTE	2^nd^ CASSETTE	Putative location of mCherry	Stable population
pCIT-mChe	5′*EtMIC1-mCitrine-*3′*EtMIC1*	5′*EtActin-mCherry-*3′*EtMIC3 (no ATG)*	non-expression	—
p5M2-mChe	5′*EtMIC1-mCitrine-*3′*EtMIC1*	5′*EtMIC2-mCherry-*3′*EtMIC3*	cytosolic	*Et*-mChe
p5M5-mChe	5′*EtMIC1-mCitrine-*3′*EtMIC1*	5′*EtMIC5-mCherry-*3′*EtMIC3*	cytosolic	—
p5M9-mChe	5′*EtMIC1-mCitrine-*3′*EtMIC1*	5′*EtMIC9- mCherry-*3′*EtMIC3*	cytosolic	—
p5M2-SP2-mChe^a^	5′*EtMIC1-mCitrine-*3′*EtMIC1*	5′*EtMIC2-ssEtMIC2-mCherry-*3′*EtMIC3*	secreted	*Et*-SP2-mChe
p5M5-SP5-mChe	5′*EtMIC1-mCitrine-*3′*EtMIC1*	5′*EtMIC5-ssEtMIC5-mCherry-*3′*EtMIC3*	secreted	*Et*-SP5-mChe^d^
p5M9-SP9-mChe	5′*EtMIC1-mCitrine-*3′*EtMIC1*	5′*EtMIC9-ssEtMIC9-mCherry-*3′*EtMIC3*	secreted	—
p5TIF-mChe^b^	5′*EtMIC1-mCitrine-*3′*EtMIC1*	5′*EtTIF-mCherry-*3′*EtMIC3*	cytosolic	—
p5TIF-SP2-mChe	5′*EtMIC1-mCitrine-*3′*EtMIC1*	5′*EtTIF-ssEtMIC2-mCherry-*3′*EtMIC3*	secreted	—
p5TIF-SP5-mChe	5′*EtMIC1-mCitrine-*3′*EtMIC1*	5′*EtTIF-ssEtMIC5-mCherry-*3′*EtMIC3*	secreted	—
p5TIF-SP9-mChe	5′*EtMIC1-mCitrine-*3′*EtMIC1*	5′*EtTIF-ssEtMIC9-mCherry-*3′*EtMIC3*	secreted	—
pmChe^c^	—	5′*EtMIC1-mCherry-*3′*EtMIC3*	cytosolic	—
p5M2-SP2-mChe-GPI^a^	5′*EtMIC1-mCitrine-*3′*EtMIC1*	5′*EtMIC2-ssEtMIC2-mCherry-gpiEtSAG1-*3′*EtMIC3*	surface	*Et*-SP2-mChe-GPI
pMIC2-mChe^a^	5′*EtMIC1-mCitrine-*3′*EtMIC1*	5′*EtMIC2-EtMIC2-mCherry-*3′*EtMIC3*	micronemes/secreted	*Et*-MIC2-mChe^d^

^a^Used to obtain stable populations: *Et*-SP2-mChe, *Et*-SP2-mChe-GPI and *Et*-MIC2-mChe; respectively.

^b^Marugan-Hernandez *et al*.^4^.

^c^Clark *et al*.^47^.

^d^Not characterised in the study.

### Fused SP2-mCherry and SP2-mCherry-GPI were found in the sporocyst cavity after oocyst sporulation but not in the parasitophorous vacuole (PV) of invaded cells

Observation of transient transfected sporozoites during *in vitro* culture provided no evidence of mCherry secretion into the PV, but the detection of SP2 targeted mCherry in vesicles (Fig. 1a panel 2) and the ability of fused SP2-mCherry-GPI to reach the sporozoite surface (Fig. 1a panel 4), both support the hypothesis that mCherry has entered the endomembrane system and would be secreted if not fused to a GPI anchor. To better evaluate secretion we generated stable populations of transgenic parasites (Table 2) expressing fused SP2-mCherry (*Et*-SP2-mChe from plasmid p5M2-SP2-mChe; Supplementary Fig. S1) or fused SP2-mCherry-GPI (*Et*-SP2-mChe-GPI, from p5M2-SP2-mChe-GPI; Supplementary Fig. S1), with non-fused mCherry as a non-secretion control (*Et*-mChe from p5M2-mChe; Supplementary Fig. S1).

**Figure 2:**
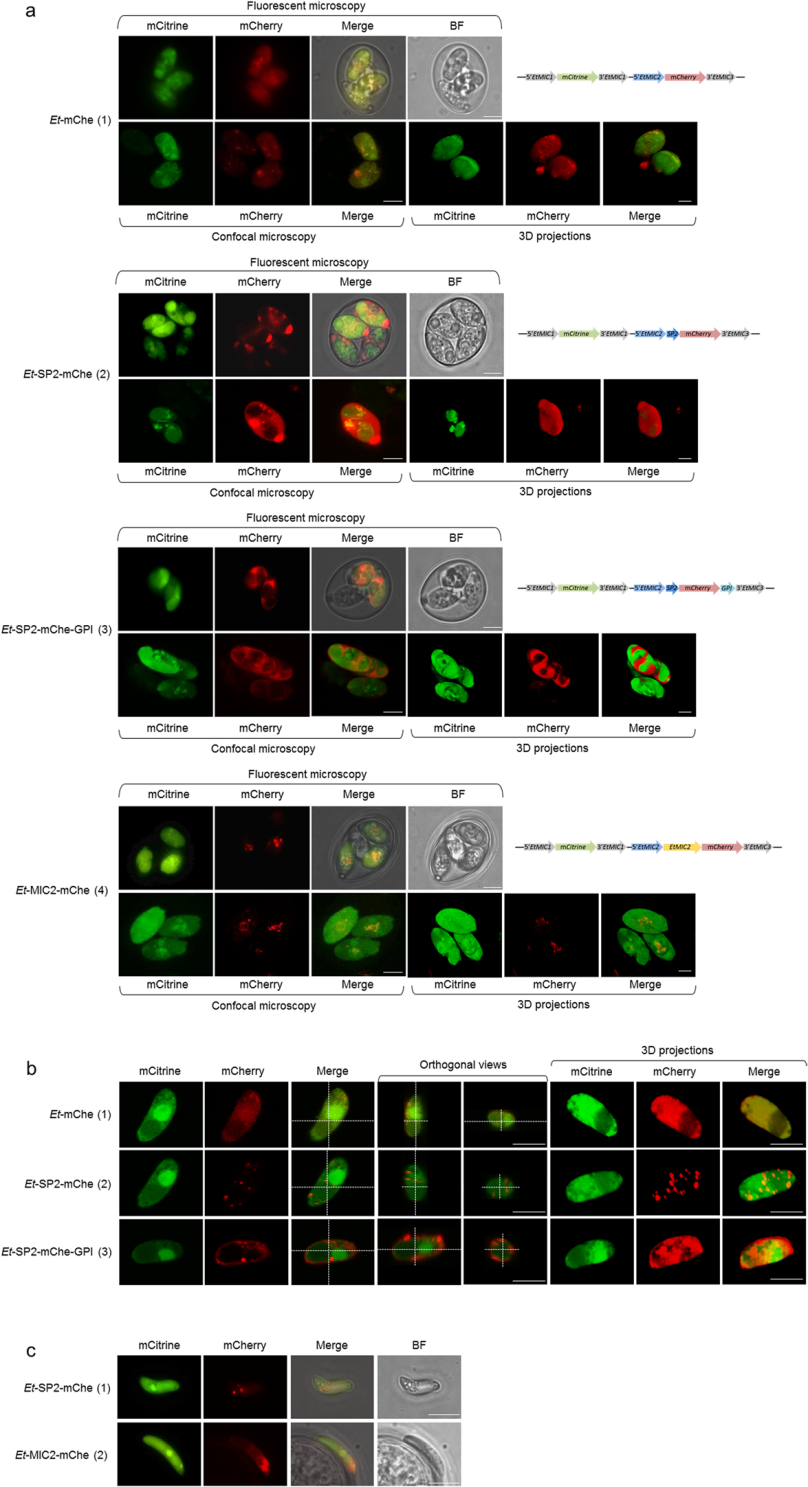
Fluorescent patterns observed in stable transgenic populations of *E. tenella* (mCitrine was always observed in the cytosol for all the populations at all different stages). (**a**) Micrographs of oocysts of the transgenic populations: fluorescent microscopy (^1^), confocal microscopy (^2^) and 3D projections. Oocysts can contain a variable number (1 to 4) of fluorescent sporocysts. (1) *E*t-mChe: cytosolic expression of mCherry; (2) *E*t-SP2-mChe: extracellular expression of mCherry, accumulated in the sporocyst cavity; (3) *E*t-SP2-mChe-GPI: uneven surface expression of mCherry; (4) *E*t-MIC2-mChe: intracellular expression of mCherry, accumulated in cytoplasmic vesicles. (**b**) Fluorescent patterns, orthogonal views (^2^) and 3D projections (^3^) of intracellular sporozoites. (1) *Et*-mChe: mCherry in the cytosol; (2) *Et*-SP2-mChe: mCherry in vesicles in the cytoplasm; (3) *Et*-SP2-mChe-GPI: mCherry on the surface with different degrees of aggregation. (**c**) Hatched sporozoites (^1^). (1) *E*t-SP2-mChe: mCherry is observed in vesicles in the anterior part of the sporozoite; (2) *E*t-MIC2-mChe: apical expression of mCherry. (^1^) Micrographs taken with DCF365FX camera using a Leica DMI3000B fluorescent microscope; (^2^) Leica Confocal microscope SP5; (^3^) 3D projections are derived from different positions along the z-axis of the confocal micrographs (z stacks) processed with Velocity software version 6.3, rotation of the images was done to show an improved view of the mCherry distribution. BF = Bright field. Bars represent 5 *µ*m.

In sporulated transgenic oocysts mCitrine, as expected, was confined to the cytosol of the sporozoites as was mCherry in *Et*-mChe (non-fused; Fig. 2a panel 1). In both of the transgenic populations with fused mCherry (*Et*-SP2-mChe and *Et*-SP2-mChe-GPI) this did not co-localise with mCitrine, providing evidence of differential targeting. In *Et*-SP2-mChe the mCherry accumulated outside of the sporozoites, within the sporocyst cavity and at the anterior pole around the micropyle (Fig. 2a panel 2). In *Et*-SP2-mChe-GPI the pattern of mCherry expression was distinctly different with accumulation around the surface of the sporozoites, but not at the anterior pole of the sporocyst (Fig. 2a panel 3).

Sporozoites purified from transgenic oocysts recovered after *in vivo* passage were allowed to invade MDBK cell monolayers. Under confocal microscopy there was no evidence of mCherry secretion into the PV by intracellular sporozoites of *Et*-SP2-mChe (Fig. 2b panel 2) or by any of the other populations (*Et*-SP2-mChe-GPI or *Et*-mChe; Fig. 2b panels 3 and 1). In a 3D projection of *Et*-SP2-mChe sporozoites, mCherry appears to accumulate in vesicles scattered throughout the cytoplasm (Fig. 2b panel 2), some of them close to the parasite membrane. In contrast, in both confocal images and 3D projections of *Et*-SP2-mChe-GPI sporozoites, mCherry was associated with the sporozoite surface with an uneven distribution (Fig. 2b panel 3) comparable to the oocysts shown in the Figure 2a (panel 3).

### mCherry was found in the supernatants of transgenic parasite populations *Et*-SP2-mChe and *Et*-SP2-mChe-GPI after hatching and only in those of *Et*-SP2-mChe-GPI during invasion

**Figure 3:**
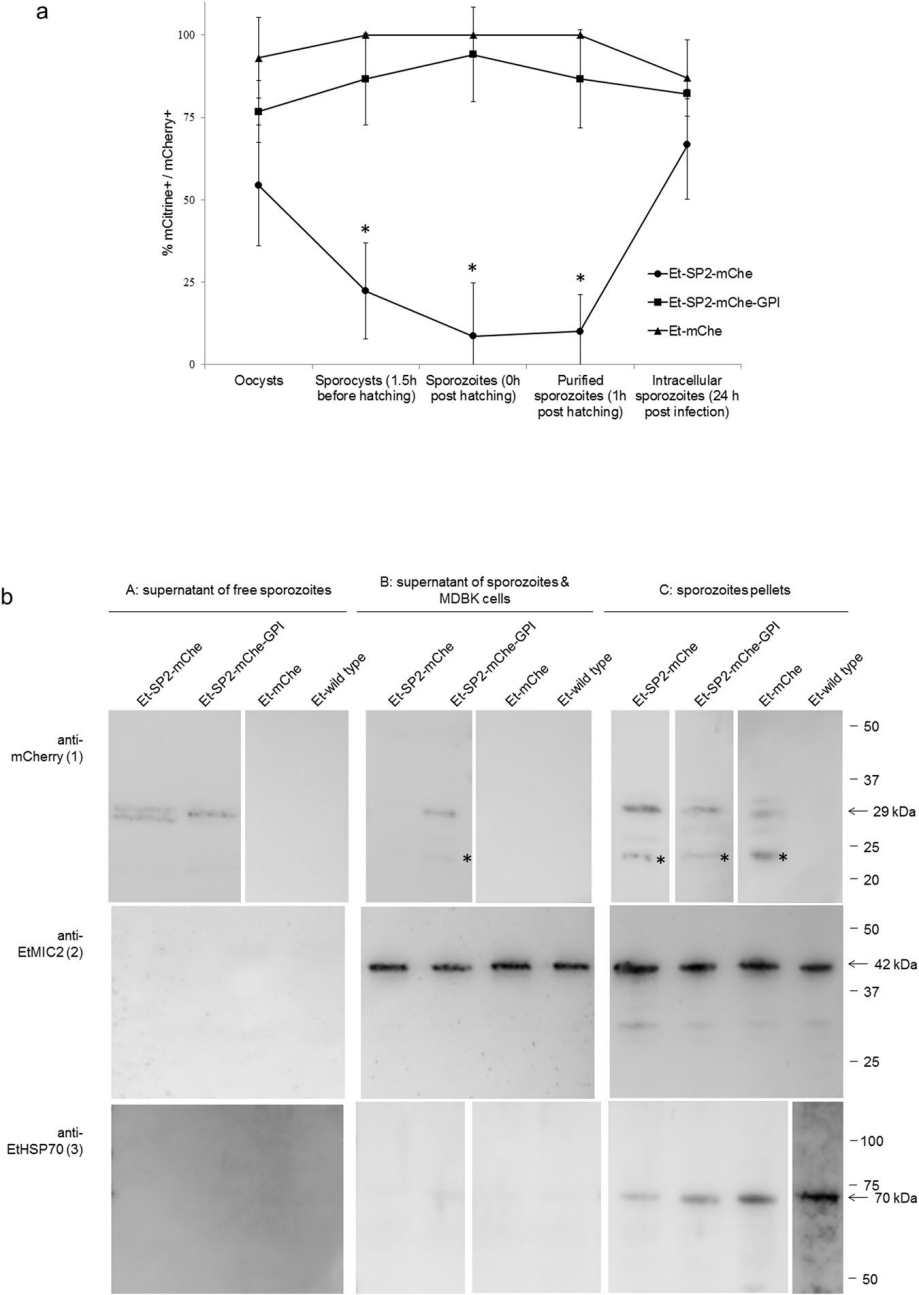
Analysis of the secretion/release of mCherry. (**a**) Proportion of double positive (mCitrine+/mCherry+) parasites at different stages of sporozoite excystation and host cell invasion. *Indicate significant differences (p < 0.05, one-way ANOVA with post hoc Bonferroni test). (**b**) Western blot analysis of sporozoites supernatants. A: supernatants of free hatched sporozoites incubated in Advanced DMEM for 30 min at 41 °C – 5% CO_2_. B: supernatants of sporozoites incubated with MDBK cells in Advanced DMEM for 1 h at 41 °C – 5%CO_2_. C: sporozoites pellets (positive controls of expression). (1) anti-mChe antibody: mCherry was detected with the expected size in the supernatants of free sporozoites (A) of *E*t-SP2-mChe and supernatant of free sporozoites (A) and incubated with MDBK cells (B) of *E*t-SP2-mChe-GPI. mCherry was detected in the sporozoites pellets (C) of all the transgenic populations (*E*t-SP2-mChe, *E*t-SP2-mChe-GPI and Et-mChe) but not in the wild type, as expected. A smaller unspecific band (*) was found in the positive samples. (2) anti-EtMIC2 antibody: endogenous EtMIC2 was detected in all the supernatants of sporozoites incubated with MDBK cells (B) and all the sporozoites pellets (C), confirming the induction of secretion of microneme proteins in the presence of the host cell. (3) anti-EtHSP70 antibody: EtHSP70 was only detected in the sporozoite pellets (C), confirming that parasites had not lysed and released their contents into the supernatants. Full length blots are presented in Supplementary Fig. S2.

Next, we defined mCherry expression and release during excystation and host cell invasion. The proportion of mCitrine/mCherry double positive parasites differed between transgenic populations. For *Et*-SP2-mChe only 54% of the mCitrine positive oocysts also expressed mCherry, in contrast to 75% of *Et*-SP2-mChe-GPI and 93% of *Et*-mChe (Fig. 3a). The proportion of double positive *Et*-SP2-mChe-GPI sporocysts remained high (86%) with no significant variation throughout sporozoite release and purification (94% and 86% respectively; Fig. 3a), supporting the suggestion that secreted mCherry was primarily retained on the sporozoite surface. All parasites from the *Et*-mChe population expressed both reporter proteins in the cytosol of sporocysts and sporozoites, before and after purification (Fig. 3a). In contrast, during sporocyst excystation *Et*-SP2-mChe sporocysts showed a significant reduction in mCherry fluorescence whilst remaining mCitrine positive (22% double positive; p < 0.05, one-way ANOVA with post hoc Bonferroni test; Fig. 3a) compared to the other two populations. Sporozoite release was accompanied by a further significant reduction in double positive *Et*-SP2-mChe parasites (p < 0.05, one-way ANOVA with post hoc Bonferroni test; Fig. 3a), representing 9% of the population after hatching and 10% after purification (approximately one hour later), suggesting that these sporozoites continued to secrete mCherry. Intracellular *Et*-SP2-mChe sporozoites assessed 24 hours after addition to MDBK cells had an increased proportion of double positives (67%) similar to levels observed in the other populations (p > 0.05, one-way ANOVA with post hoc Bonferroni test; Fig. 3a). While these observations correlate with possible secretion/release of the mCherry reporter, it was difficult to directly visualise secretion from sporozoites since the fluorescent protein signal would have been diluted in the culture supernatant below the limit of microscopic detection. Thus, culture supernatants were recovered and analysed for the presence of mCherry by Western blotting (Fig. 3b).

In the first secretion assay where excysted sporozoites were incubated in advanced DMEM (without serum) mCherry was readily detected in culture supernatants from both mCherry-fused transgenic populations (Fig. 3b A panel 1). In the second assay, when sporozoites were incubated with MDBK cells, mCherry was detected only in the supernatants of *Et*-SP2-mChe-GPI (Fig. 3b B panel 1). These results correlate with the previous observations in which mCherry in the *Et*-SP2-mChe population faded from sporozoites over time (only 10% were mCherry positive, Fig. 3a), possibly because it was being secreted and therefore depleted whilst the parasites were incubated with the cells. However, the profile observed for *Et*-SP2-mChe-GPI was different, with mCherry present in supernatants collected before and after incubation with cells. No mCherry was found in any of the supernatants of *Et*-mChe (non-fused, cytosolic) or wild type *E. tenella* sporozoites (Fig. 3b panel 1).

When the same supernatants were analysed for the presence of EtMIC2, this was detected only when sporozoites were exposed to host cells (as expected for a microneme protein; Fig. 3b B panel 2) but not immediately after hatching (Fig. 3b A panel 2). The lack of EtHSP70 in any of the supernatants (Fig. 3b panel 3) indicated that detection of mCherry was not due to inadvertent sporozoite lysis. We used pellets of sporozoites representing the different populations as positive controls for the presence of each protein target (mCherry, EtMIC2 or EtHSP70, Fig. 3b C).

### mCherry in *Et*-SP2-mChe parasites showed a different pattern than in parasites containing the complete EtMIC2 coding sequence fused to mCherry (*Et*-MIC2-mChe)

In addition to its secretion into the sporocyst cavity whilst sporozoites were within *Et*-SP2-mChe oocysts (Fig. 2a panel 2) we found secretion of mCherry into supernatants by free sporozoites (Fig. 3b A panel 1). In the absence of specific targeting signals we would not expect this molecule to be secreted via the microneme organelles, and although the vesicles observed in the intracellular sporozoite do not initially correspond to these organelles, we generated transgenic parasites expressing full-length EtMIC2 (containing therefore all microneme targeting elements) fused to mCherry (*Et*-MIC2-mChe; Table 2; Supplementary Fig. S1) to compare the red fluorescent pattern. In these parasites the distribution of mCherry was distinct from that seen in oocysts or sporozoites of *Et*-SP2-mChe or *Et*-SP2-mChe-GPI (Fig. 2a panels 2 and 3), with mCherry retained entirely inside the sporozoites (but not in the cytosol; Fig. 2a panel 4). Due to sporozoite folding within the sporocyst it was difficult to describe the precise location of mCherry in the *Et-*MIC2-mChe oocysts; however an apical position within sporozoites looked plausible. In hatched *Et*-MIC2-mChe sporozoites it was indeed clear that mCherry was concentrated at the apical pole in the expected position of the micronemes (Fig. 2c panel 2) which was distinct from mCherry in *Et*-SP2-mChe sporozoites (Fig. 2c panel 1), which as described above was seen in vesicles.

This, together with the different timing in secretion seen in the Western blots (mCherry *vs*. EtMIC2 in Et-SP2-mChe, Fig. 3b) led us to conclude that fused SP-mCherry is most likely transported through the secretory pathway without the involvement of the micronemes.

### mCherry accumulates inside sporozoites during intracellular development of *Et*-SP2-mChe and exhibits a surface distribution in *Et*-SP2-mChe-GPI

**Figure 4:**
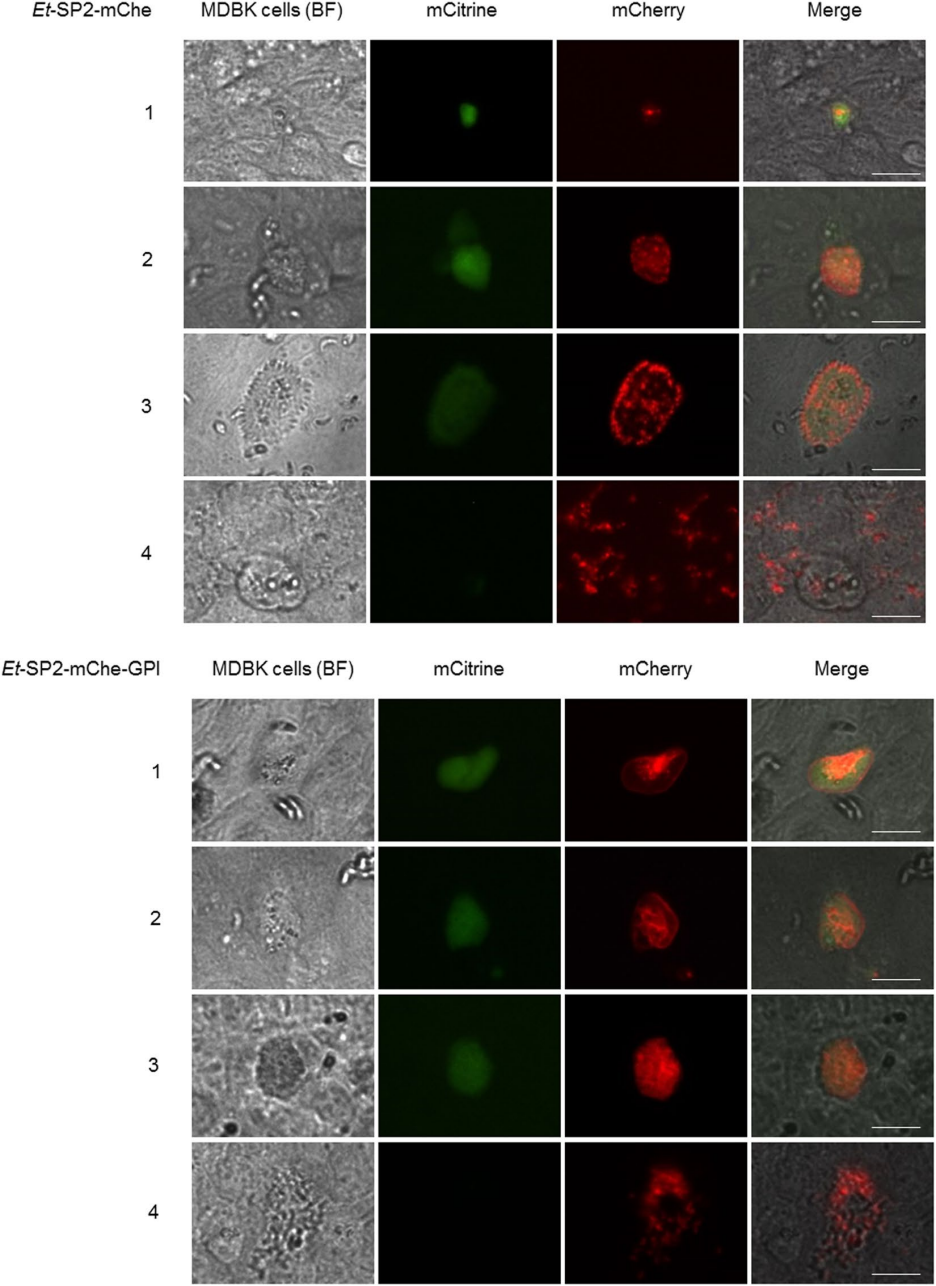
*In vitro* intracellular development of *Et*-SP2-mChe and *Et*-SP2-mChe-GPI after incubation in MDBK cells for 48 h (41 °C, 5% CO_2_). Panels 1 and 2: schizont development; mCherry accumulated in vesicles in *Et*-SP2-mChe whereas it was membrane anchored in *Et*-SP2-mChe-GPI. Panels 3 and 4: first generation merozoite formation and release; mCherry was intracellular in the newly formed merozoites in *Et*-SP2-mChe, while appearing to be membrane anchored in nascent merozoites in *Et*-SP2-mChe-GPI. mCitrine almost completely vanished in both populations. BF = bright field. Bars represent 20 *µ*m.

In short term cultures we were not able to observe mCherry secretion/release into the PV once the parasite became intracellular. For better characterisation of possible secretion into the PV, sporozoites from stable transgenic populations of *Et*-SP2-mChe and *Et*-SP2-mChe-GPI were used to infect monolayers of MDBK cells and allowed to develop through schizogony and produce 1^st^ generation merozoites. At 24 hours, intracellular sporozoites expressed both reporters with exactly the same patterns described before (Fig. 2b panels 2 and 3) with no detection of mCherry within the PV for either population. After 48 hours many intracellular sporozoites had developed into schizonts and for both populations mCitrine showed a continuing cytosolic distribution (Fig. 4 panels 1 to 3) until it disappeared in the released merozoites (Fig. 4 panel 4). In *Et*-SP2-mChe parasites mCherry expression was intracellular (Fig. 4 *Et*-SP2-mChe panels 1 to 4) and as the schizonts enlarged and merozoites were released the observed pattern was consistent with mCherry being expressed within vesicles in the daughter merozoite (Fig. 4 *Et*-SP2-mChe panels 3 and 4). In *E*t-SP2-mChe-GPI parasites, mCherry was distinctly attached to the schizont outer and internal membranes as schizonts enlarged (Fig. 4 *Et*-SP2-mChe-GPI panels 1 and 2) then finally reorganised into the newly formed merozoites (Fig. 4 *Et*-SP2-mChe-GPI panels 3 and 4). Again, we did not see evidence of secretion/release of mCherry into the PV in any of the stages; mCherry was always confined within the schizont structure acquiring different distributions for each transgenic population throughout development.

## Discussion

The mechanisms governing intracellular trafficking in apicomplexan parasites are not fully understood and differ between genera, however there are a number of studies that describe how specific peptide sequences influence the final destination of proteins. In *T. gondii* it has been postulated that the addition of an N-terminal SP without any other targeting signals results in constitutive secretion of the protein *via* dense granules^24, 27^. Moreover, the addition of a GPI anchor re-routes proteins to the parasite surface^24^. A fusion of a SP with a fluorescent reporter has been done before in *E. tenella* ^3^; however precise localization and potential secretion of the fused protein was not clearly defined. In this study we generated and characterised *E. tenella* parasites expressing mCherry fused to the SP of EtMIC2 (*Et*-SP2-mChe) and also with the addition of the GPI-anchor of EtSAG1 (*Et*-SP2-mChe-GPI), finding similarities but also some differences to what has been reported in *Toxoplasma*.

Under control of the 5′*EtMIC2* promoter, mCherry was expressed in the cytosol of sporozoites and the addition of SP2 to its N-terminus in *Et*-SP2-mChe parasites resulted in mCherry translocation into the parasite endomembrane network, evidenced by the observation of red vesicles scattered throughout the sporozoite cytoplasm. Secretion of mCherry was observed in the sporocyst cavity of oocysts and into culture supernatants by released sporozoites, in which intracellular mCherry gradually disappeared, likely secreting until exhausted. Nonetheless, in contrast to what has been described for *Toxoplasma* tachyzoites, mCherry was never observed within the PV in invaded cells. The addition of a GPI-anchor at the C-terminus of the SP2-mCherry fusion modified the destiny of the reporter, re-locating it to the surface of the sporozoite; however, this surface distribution was non-homogenous with some areas containing higher concentrations of the reporter. mCherry was also found in culture supernatants of released sporozoites, nevertheless the reporter did not disappear from sporozoites before invasion, which leads us to believe that detection in the supernatants is probably due to cleavage of the protein from the surface, rather that its release by secretion.

We can hypothesise that addition of SP2 allowed mCherry trafficking through the constitutive secretory pathway as was demonstrated for *Toxoplasma*. However, once the sporozoite invades the host cell and becomes intracellular this process appears to be under tighter regulation as mCherry secretion was not detected into the PV, suggesting that *E. tenella* sporozoites exert specific control over what is secreted at this point of the lifecycle. This is different to what has been described for tachyzoites of *T. gondii*, where constitutive secretion of soluble proteins into the PV *via* the dense granules is considered a ‘default’ route. Nonetheless, our observations correlate with what was described in *Toxoplasma* sporozoites in which some dense granule proteins are retained within cytoplasmic granules instead of being secreted into the PV1, a temporary vacuole produced before the PV2 in which the parasite would develop into tachyzoites^28^. The formation of transient PV different to the classic moving junction-mediated PV^29^ in different host tissues before reaching the liver has been also reported in *Plasmodium* ^30, 31^. In addition, invasion through breaching of host cell plasma membranes by *Eimeria bovis* and *E. tenella* has also been described^32, 33^. There are many differences in endogenous development of coccidian sporozoites and tachyzoites (schizogony *vs*. endodyogeny^34^) including details of PV structure, composition and lifespan that could require different control of secretion at this stage. Moreover, the presence of dense granules as an independent organelle in *Eimeria* spp. has been questioned due to the low number of dense granule gene orthologues found in their genomes^35^ and a lack of definitive microscopic visual evidence. Also in agreement with this, a downregulation in dense granules protein (GRA) expression has been reported in *T. gondii* merozoites^36^ in comparison to the tachyzoites. Thus our data on sporozoites of *E. tenella* provides some support for the idea that the gut stages of coccidian development may utilise an alternative pathway for the secretion of soluble proteins.

Our data indicate that this alternative route is unlikely to be through the micronemes. We observed differential localization of mCherry in *Et*-SP2-mChe parasites compared to *Et*-MIC2-mChe parasites as well as differences in the timing of mCherry secretion compared to the endogenous EtMIC2 microneme protein. Moreover, when Huang *et al*.^3^ fused the SP of EtMIC1 to EYFP in *E. tenella* it led to secretion into the sporocyst cavity but apparently not into the PV of infected host cells, which agrees perfectly with our observations. In contrast, when the complete coding sequence of *Toxoplasma* TgGRA7 was fused to YFP and expressed in *E. tenella* ^37^ it was detected in the PV and sporocyst cavity, suggesting that the targeting information contained in the coding sequence of TgGRA7 can direct the protein to the dense granules or equivalent vesicles specifically programmed to be secreted into the PV. Therefore, in *E. tenella* secretion into the PV would require the presence of specific signals in PV proteins, because the ‘default’ secretory pathway is tightly controlled when inside the host cell. It remains to be determined what type of vesicles and/or storage organelles are associated with this pathway in the absence of classical dense granules. In our observations vesicles containing mCherry could be a dense granule-like vesicle, although the regulation of the secretion seems to differ from that described for *Toxoplasma* ^24^. To further investigate the actual location of these vesicles, a series of co-localization studies with markers of different structures (ER/Golgi) and organelles (micronemes/rhoptries) would be of great interest.

It is also relevant that expression of the GPI anchor could re-locate the reporter onto the surface of sporozoites; however, mCherry was also detected in the supernatants of incubated free sporozoites. In other Apicomplexa it has been reported that the targeting of proteins can be modified depending on the promoter strength and timing^13, 38^. Here, overexpression mediated by the 5′*EtMIC2* promoter region compared to the usual levels of *E. tenella* SAG proteins produced in the sporozoite stage (transcription from 10 to 127,000 times higher for EtMIC2, 15 times higher than for the major surface antigen EtSAG1^35^) could be saturating the anchoring sites and causing a release of mCherry into the media, which would explain why it was present in the supernatants. In addition, mCherry presence in the supernatants during the course of invasion of the host cells could also be explained by capping of the GPI-anchored proteins from the parasite surface during the cell invagination process^39^. In *T. gondii*, GPI anchors appear to contain the targeting signals to deliver proteins to the parasite membrane, although the specific route by which they reach the surface is unknown^24, 40^. Conversely, not all GPI-anchored proteins traffic to the membrane since TgSUB1, which is GPI-anchored, is stored in micronemes^41^. In our study, the addition of the GPI anchor re-directed the reporter to the surface with the presence of vesicles only visible toward the apical end.

The behaviour of the mCherry reporter protein during intracellular development of *Et*-SP2-mChe and *Et*-SP2-mChe-GPI parasites was very informative. In both populations the intracellular vesicles appear to fuse and concentrate into a single one in the early stages. In later stages many vesicles were detected in *Et*-SP2-mChe, which could be derived from the previous one or be newly formed; whereas a clear membrane association was observed for *Et*-SP2-mChe-GPI. Further study of these transgenic populations could help in understanding these secretion vesicles and membrane biogenesis during the course of the parasites endogenous development.

We failed to visualise SP5-mCherry under its endogenous promoter (5′*EtMIC5*) in transient transfection, which was unexpected as the promoter proved to be functional and EtMIC5 is a well characterised microneme protein with a functional SP (whereas EtMIC9 is present in the databases, but not tested). Moreover, when SP5-mCherry was expressed under 5′*EtTIF* detection was insufficient to assign localization. We speculated this may be due to early secretion into the supernatant before invasion, thus, to get any detection of mCherry we generated stable transgenic oocysts with p5M5mChe and interestingly, red fluorescence was found in a very low proportion of mCitrine expressing oocysts (5%) and showing exactly the same pattern as that seen in oocysts of *Et*-SP2-mChe.

Further work has to be done with *Eimeria* in order to define the precise mechanisms that govern cell trafficking and clarify the similarities and differences with *Toxoplasma*. It is important to highlight the differences of working with *E. tenella* in relation of *T. gondii*; the inability of *E. tenella* sporozoites to grow productively in cell culture, the difficulties in obtaining clonal lines and the lack of molecular tools for the generation of knock outs or regulation of gene expression makes the study of these mechanisms very difficult. Nonetheless, *E. tenella* is an extremely important pathogen of domestic livestock as well as the best available model for studying the sporozoite stage of coccidian parasites. A better characterisation of the composition of different invasion compartments, including those that mediate regulated and non-regulated secretion, as well as the endosomal-like system and the plant-like vacuole^10, 42^ would be of great importance to understand the secretory pathway in the enteric stages of coccidia. Finally, regardless of the mechanisms by which proteins traffic within *Eimeria*, these novel transgenic parasites are able to secrete foreign antigens or display them on the cell surface, and it will be interesting to determine whether this could lead to improved antigen recognition by the chicken immune system as has been described before for *Toxoplasma* and other parasites^6, 43^.

## Materials and Methods

### Parasites and birds

Oocysts of the *E. tenella* Wisconsin strain^44^ were propagated in 3-to-4 week-old White Leghorn chickens (supplied by MSD Animal Health, produced and reared under specific pathogen free conditions) using routine methods^45^. Sporozoites were excysted and purified following established protocols^46^ and used for transfection followed by both *in vivo* and/or *in vitro* infection^4^. *In vitro* infections were performed in MDBK (NBL-1; ECACC-Sigma-Aldrich, Salisbury, UK) cell monolayers grown in 24-well plates (0.3 × 10^6^ cells per well) and incubated at 41 °C, 5% CO_2_ in Advanced DMEM (Gibco, Leicestershire, UK) supplemented with 2% foetal bovine serum (FBS; Sigma, Suffolk, UK) and 100 U penicillin/streptomycin (Fisher, Leicestershire, UK).

### Plasmid constructs for expression of the fluorescent reporter mCherry

The plasmid pCIT_CjaA^5^ was used to generate a new plasmid, pCIT-mChe that contained two fluorescent reporters (Supplementary Fig. S1). The first reporter, *mCitrine*, was situated downstream of the 5′*EtMIC1* promoter region and expressed as a soluble cytosolic protein in the absence of targeting signals; used throughout this work to detect and select transgenic parasites by FACS. The second reporter, *mCherry*, was cloned without its start codon to replace *cjaA* in pCIT_CjaA using primers incorporating *Xba* I and *Bgl* II restriction sites. The specific location of this expressed reporter varies according to various additional sequences fused to it. All plasmids used or generated in this study are listed in Table 2 and primers are listed in Supplementary Table S1.

The region upstream of *mCherry* (5′*EtActin*) in pCIT-mChe was replaced by ~1 Kb of 5′ upstream sequences of genes *EtMIC2*, *EtMIC5* or *EtMIC9*, (Supplementary Fig. S1) or by these same 5′ upstream sequences plus their corresponding signal peptide sequences (*sp*) (Supplementary Fig. S1) using primers including *Bam* HI and *Xba* I restriction sites. The constitutive promoter region 5′*EtTIF* ^4^ was then cloned upstream of each *sp* fused to *mCherry* (Supplementary Fig. S1) replacing the 5′*EtMIC* promoter regions. Briefly, the 5′*EtTIF* region was amplified from p5TIF-mChe^4^ plasmid (Supplementary Fig. S1) using primers including *Bam* HI and *Cla* I restriction sites and cloned into pCIT-mChe plasmid (Supplementary Fig. S1) digested by the same endonucleases. Next, each *sp* fused to *mCherry* (*spEtMIC2-mChe*, *spEtMIC5-mChe*, *spEtMIC9-mChe*) was amplified from the respective plasmid with primers including *Cla* I and *Bgl* II restriction sites and cloned downstream of *5*′*EtTIF*. A single cassette plasmid containing *mCherry* under control of the promoter region 5′*EtMIC1* ^47^ was also included in the study as a control in the transient transfection studies (Supplementary Fig. S1).

Sequences encoding the GPI signal anchor from *E. tenella* surface antigen 1 (*gpiEtSAG1 –* AAGGGCGGAGTTTCTCCAGTCGTCCCTTCAGTAGCCCTCATCTCTGCGGCGGTCATCTCCGCTTTCGCTCTCTTTTAG) were ligated to the C-terminus of *spEtMIC2-mChe* (*spEtMIC2 –* ATGGCTCGAGCGTTGTCGCTGGTCGCTTTGGGCTTGCTTTTTTCCCTTCCTCCAAGCTCAGCCGTT) in order to attach secreted protein to the surface of the parasite. For that, the *mCherry* coding sequence was excised then re-cloned into the same site removing the endogenous stop codon using primers including *Xba* I and *Bgl* II restriction sites. The amplified *gpi* sequence was cloned downstream of *mCherry* using primers containing *Bgl* II restriction sites; the correct orientation of the encoded *gpi* signal anchor was confirmed by sequencing (Supplementary Fig. S1). The complete coding sequence of *EtMIC2* without its endogenous stop codon was cloned into the *Xba* I site of pCIT-mChe (Supplementary Fig. S1) to provide a fused EtMIC2-mCherry as control for micronemal location.

All PCR products were sub-cloned into pGEM®-T Easy Vector (Promega, Hampshire, UK) and digested by the respective restriction enzymes (NEB, Hertfordshire, UK) for subsequent ligation by T4 ligase (Promega) into the corresponding plasmid. The correct sequence of each new plasmid was confirmed by sequencing (GATC Biotech, Konstanz, Germany) and assembly using CLC Main Workbench v6.7.1 (CLC Bio, Katrinebjerg, Denmark). Final plasmids were prepared for transfection using an EndoFree Plasmid Maxi Kit (Qiagen, West Sussex, UK), digested for linearization with *Psi* I, precipitated in ethanol-sodium acetate and quantified by NanoDrop (Thermo, Massachusetts, USA).

### Analysis of mCherry location in transiently transfected *E. tenella* sporozoites

Restriction enzyme-mediated integration (REMI) transfection into *E. tenella* sporozoites was carried out in 16-well strips using the programme EO114 of the Nucleofector 4D (Lonza, Basel, Switzerland) as described previously^4^. For each plasmid, 1 × 10^6^ sporozoites were used per well together with 10 µg of plasmid and 6 U of *Psi* I in Lonza buffer P3; all experiments were done in triplicate. After the shock, sporozoites were diluted in RPMI medium (Sigma) and left for 20 minutes at room temperature before being added to a newly confluent monolayer of MDBK cells. Sporozoite survival was estimated by counting a sample in 0.02% Trypan blue dye (Invitrogen, California, USA). Sporozoites and cells were incubated at 41 °C for 4 hours, at which time the medium containing uninvaded parasites was removed and replaced with fresh RPMI.

At 24 hours post-infection cells were examined by fluorescence microscopy (Leica DMI3000B). The cytosolic mCitrine (emission peak 527) allowed identification of sporozoites expressing transgenes, whereas mCherry (emission peak 610) was used to provide evidence for or against the functionality of the different promoter regions tested as well as the ability of SP and GPI anchors to direct protein trafficking through the secretory pathway and/or to attach it to the parasite surface. Micrographs were taken using a digital camera (40x - brightness 10% - gain 1.0%; DCF365FX; Leica Microsystems, Milton Keynes, UK) and images were analysed using Image J (NCBI, http://rsb.info.nih.gov/ij/). Thresholds were set to create regions of interest containing fluorescent sporozoites and used to analyse parasites in original images producing relative intensity values between 0 (pure black) and 255 (pure white). The experiments were performed in triplicate analysing *ca*. 25 sporozoites per condition in each replicate. Data of the relative intensity were analysed by a one-way ANOVA with post hoc Bonferroni test (SPSS Statistics 22, New York, USA).

### Obtaining stable transgenic populations and characterization of mCherry location

To obtain stable transgenic populations, sporozoite transfections were carried out as described above using 4 wells for each plasmid which were then pooled and used to infect 4 chickens via the cloacal route (0.75 × 10^6^ initial transfected sporozoites per bird). Oocysts were harvested one week later, sporulated and used for a subsequent *in vivo* passage after enrichment of the population for fluorescent parasites by FACS (BD FACS Aria^TM^ III^4^).

Micrographs of sporulated oocysts of the transgenic populations expressing mCherry were collected using the fluorescent microscope DMI3000B – DCF365FX (Leica Microsystems) and/or the SP5 confocal microscope (Leica Microsystems) using Leica Application Suite advanced fluorescence software version 2.6 in order to determine the localization of the mCherry protein (2D images). Image processing for 3D projections was performed using the software Velocity version 6.3 form different positions along the z-axis of the confocal micrographs (z stacks) (PerkinElmer, Beaconsfield, UK).

### *In vitro* infections and secretion assay

Sporulated oocysts from passaged transgenic populations that expressed mCherry (without enrichment by FACS) and wild type oocysts (*Et*-wt) were cracked and hatched as described above in order to release sporozoites. Free sporozoites were purified in DE-52 columns^46^ and added to MDBK cells monolayers or to empty wells and then incubated in advanced DMEM-2% FBS for 48 hours at 41 °C until the observation of schizogony and merozoite formation/release. The presence of both reporters (mCitrine/mCherry double positive) along the excystation process was followed and quantified by fluorescence microscopy (Leica DMI3000B) to monitor possible changes. Counts of *ca*. 150 parasites for green and/or red fluorescence per stage: oocysts, sporocysts, excysted sporozoites, purified sporozoites and intracellular sporozoites. Data were analysed by a one-way ANOVA with post hoc Bonferroni test (SPSS Statistics 22).

To analyse secreted proteins two types of secretion assays were performed. In the first, 10^6^ freshly hatched sporozoites were incubated in 100 µl advanced DMEM for 30 minutes at 41 °C – 5%CO_2_. In the second, 10^6^ freshly hatched sporozoites were added in 100 µl advanced DMEM-2% FBS to newly confluent monolayers of MDBK cells (representing a multiplicity of infection of ~ 4:1) and incubated for one hour (41 °C – 5%CO_2_ **)**. In both assays the supernatants were recovered and analysed by Western blot.

### SDS-polyacrylamide gel electrophoresis (PAGE) and Western blot

Supernatants from *in vitro* secretion assays and sporozoites pellets of each population were electrophoresed and transferred to nitrocellulose membranes following procedures described previously by Marugan-Hernandez *et al*.^4^. Membranes were incubated for one hour with anti-mouse mCherry (1/100; Abcam, Cambridge, UK), anti-rabbit EtMIC2 (secreted; 1/200^48^) or anti-rabbit EtHSP70 (cytosolic; 1/200^49^) for one hour, then washed and incubated for another hour with goat anti-mouse or anti-rabbit IgG antibody HRP conjugate (1/1000; Merck Millipore, Hertfordshire, UK), as appropriate. Washes and development by chemiluminiscence were done as previously described^4^.

### Ethical statement

This study was carried out in strict accordance with the Animals (Scientific Procedures) Act 1986, an Act of Parliament of the United Kingdom. All animal studies and protocols were approved by the Royal Veterinary College and/or MSD Animal Health Ethical Review Committees and the United Kingdom Government Home Office under specific project licence. The laboratory work involving GMO was conducted under authorization GM9708.1, administered by the UK Health and Safety Executive. The animal facilities for GMO are classified derogated containment level 3.

## Electronic supplementary material

Supplementary Info
