# Supplementary material for: Eimeria tenella protein trafficking: differential regulation of secretion versus surface tethering during the life cycle
Source: Sci Rep. 2017 Jul 4;7:4557. doi: 10.1038/s41598-017-04049-1 (PMC5496917; doi:10.1038/s41598-017-04049-1)
Supplement: Supplementary file 1 — Supplementary Info [file 41598_2017_4049_MOESM1_ESM.pdf]

***Eimeria tenella* protein trafficking: differential regulation of secretion versus surface tethering during the life cycle**

Marugan-Hernandez, V*., Long, E., Blake, D., Crouch, C., Tomley, F.

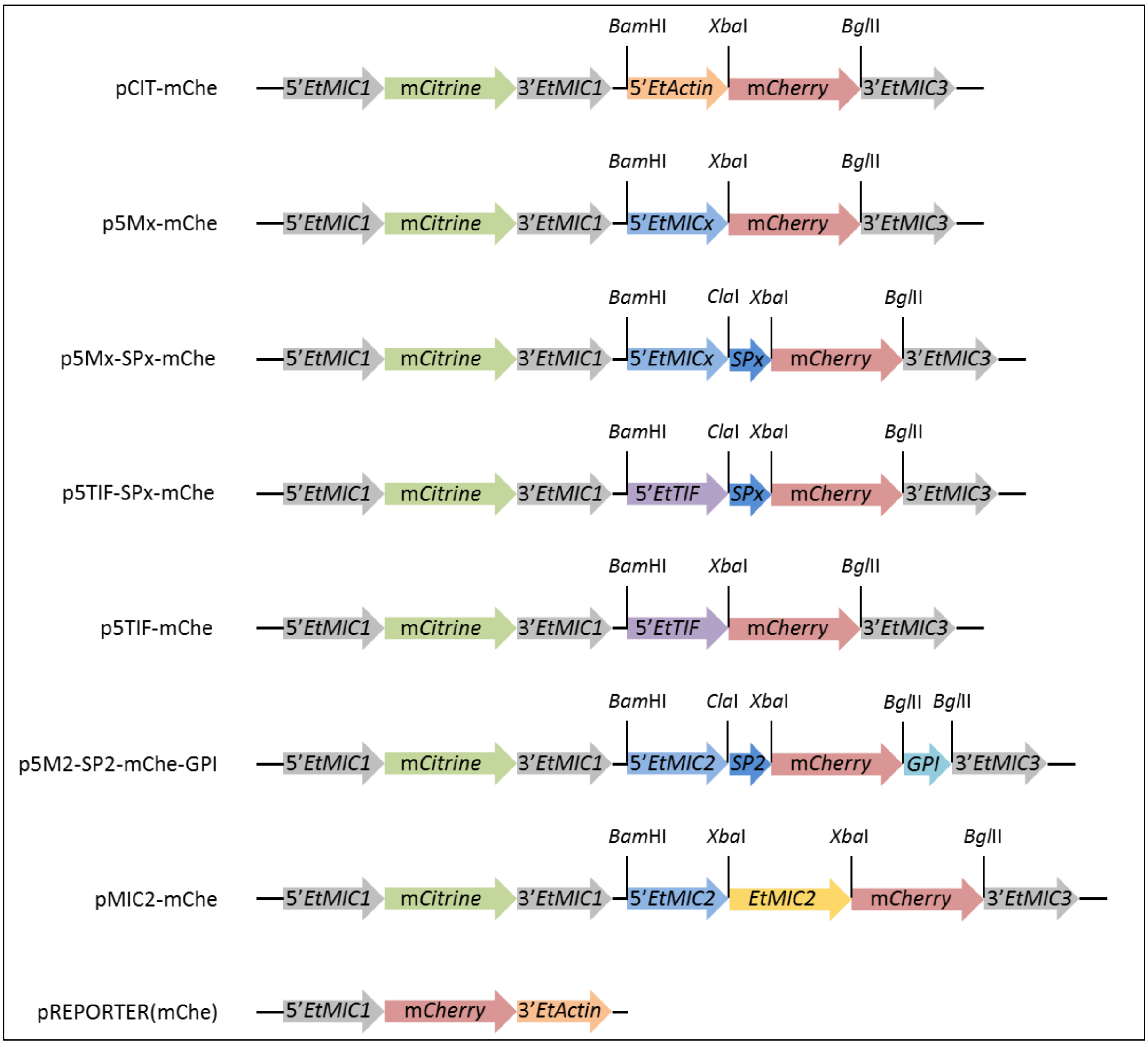

Figure S1. Plasmid constructs developed and used for the genetic complementation of *E. tenella*. All plasmids except pREPORTER(mChe) contain a double cassette: *mCitrine* – green arrow – flanked by 5'*EtMIC1* and 3'*EtMIC1* – grey arrows – and *mCherry* – red arrow – flanked by 3'*EtMIC3* – final grey arrow – are constant for all plasmids whilst the 5' promoter region of *mCherry* is exchanged. pCIT-mChe: double cassette backbone plasmid with *mCherry* flanked by 5'*EtActin* – orange arrow –; p5Mx-mChe: *mCherry* under the control of 5' microneme protein upstream regions (5Mx =

5’*EtMIC2*, 5’*EtMIC5* or 5’*EtMIC9*) – blue arrow –; p5Mx-SPx-mChe: plasmid p5Mx-mChe with microneme protein signal peptides (SPx = *spEtMIC2*, *spEtMIC5* or *spEtMIC9*) – dark blue arrow – fused at N-terminus of *mCherry*; p5TIF-SPx-mChe: *mCherry* under the control of 5’*EtTIF* upstream regions – purple arrow – with microneme protein signal peptides (*spEtMIC2*, *spEtMIC5* or *spEtMIC9*) – dark blue arrow – fused at N-terminus of *mCherry*; p5TIF-mChe: plasmid p5TIF-SPx-mChe without the microneme protein signal peptides fused at N-terminus; p5M2-SP2-mChe-GPI: *mCherry* under the control of *5’EtMIC2* (5M2) – blue arrow – with *spEtMIC2* (SP2) – dark blue arrow – fused at N-terminus of *mCherry* and the glycophosphatidylinositol (GPI) signal anchor of *EtSAG1* – pale blue arrow – fused at C-terminus of *mCherry*; pMIC2-mChe: complete *EtMIC2* coding sequence – yellow arrow – (including *spEtMIC2*) fused to *mCherry* under the control of 5’*EtMIC2* – blue arrow; pREPORTER(mChe): single cassette plasmid with *mCherry* [47] surrounded by *5’EtMIC1* – grey arrow – and 3*’EtActin* – orange arrow –.

Table S1. Primers used for plasmid construction in support of experimental *Eimeria tenella* transfection.

Name	Sequence
Fw-mChe (noATG)	GCTCTAGAGCGACCATGGTGA
Rv-mChe	GAAGATCTCTACTCTGCTGCAAACC
Fw-5’Mic2-BamHI	CGGGATCCAGAGTATTTGCTTCTGGCG
Rv-5’Mic2-XbaI	GCTCTAGACATTTTGAATGTGAA
Rv-5’Mic2-SP2-XbaI	GCTCTAGAAACGGCTGAGCTTG
Fw-5’Mic5-BamHI	CGGGATCCAACCATTTCCCCTTTAA
Rv-5’Mic5-XbaI	GCTCTAGACATTGCTTAGAGATA
Rv-5’Mic5-SP5-XbaI	GCTCTAGACCAGGCCTCTGTTCCAT
Fw-5’Mic9-BamHI	CGGGATCCCCCTGTATCAAAGTTTCATA
Rv-5’Mic9-XbaI	GCTCTAGACATCGACAGTAAGGT
Rv-5’Mic9-SP9-XbaI	CTCTAGACGGGCCAGACCCAA
Fw-5’TIF-BamHI	CGGGATCCACACCCAAAACTAAAGG

Rv-5’TIF-ClaI	CCATCGATTTGAGACATCTACAC
Fw-SP2mChe-ClaI	CCATCGATAAAATGGCTCGAGCG
Fw-SP5mChe-ClaI	CCATCGATATGGGGCGTATCCGG
Fw-SP9mChe-ClaI	CCATCGATATGTACACTTGTTGC
Rv-mChe-BglII	AAAGATCTCTACTCT
Fw-mChe-XbaI	GCTCTAGAATGGCGACCATGGTG
Rv-mChe(-TAG)-BglII	AAAGATCTCTCTGCTGCAAACCTCGACC
Fw-GPI-BglII	TTAGATCTAAGGGCGGAGTTTCTCCAG
Rv-GPI-BglII	GCAGATCTCTAAAAGAGAGCGAAAGCGG
Fw-Mic2-XbaI	TCTAGAGCTCGAGCGTTGTCGCT
Rv-Mic2-XbaI	TCTAGAGGATGACTGTTGAGTGTCACTC
Fw-vvVP2-XbaI	GCTCTAGAACAAACCTGCAAG
Rv-vvVP2-BglII	GAAGATCTTCTTACCTCCTTATAGCCCGGA
Fw-gI-XbaI	GCTCTAGAGCATCGCTACTTGGAA
Rv-gI-BglII	GAAGATCTTCTCACATTTTTATTGAGTCGGGC

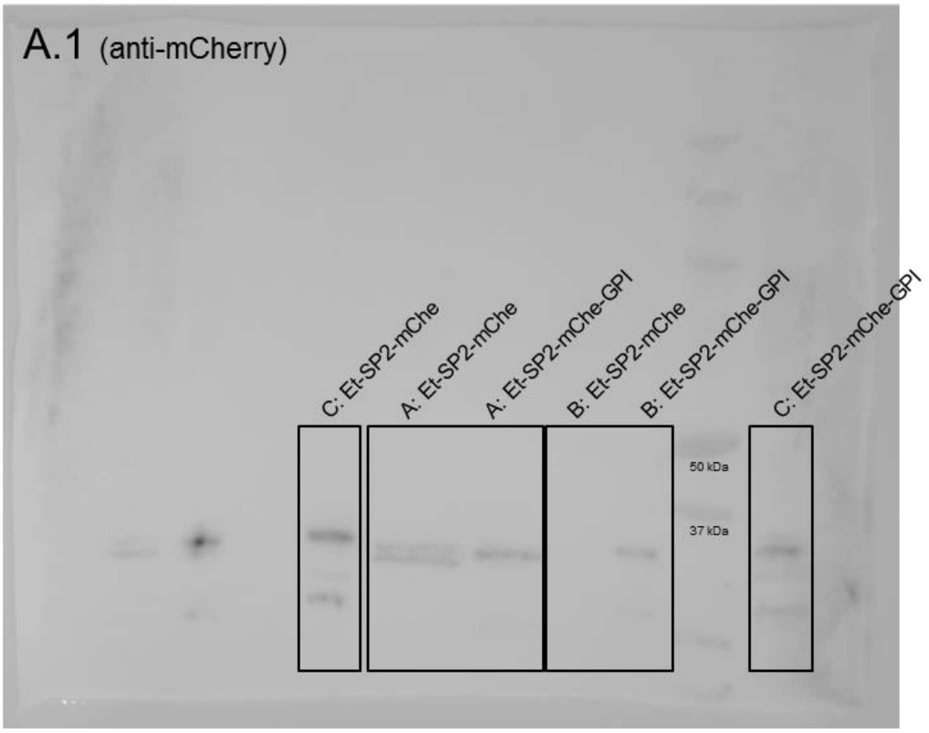
A.1 (anti-mCherry)
C: Et-SP2-mChe
A: Et-SP2-mChe
A: Et-SP2-mChe-GPI
B: Et-SP2-mChe
B: Et-SP2-mChe-GPI
C: Et-SP2-mChe-GPI
50 kDa
37 kDa

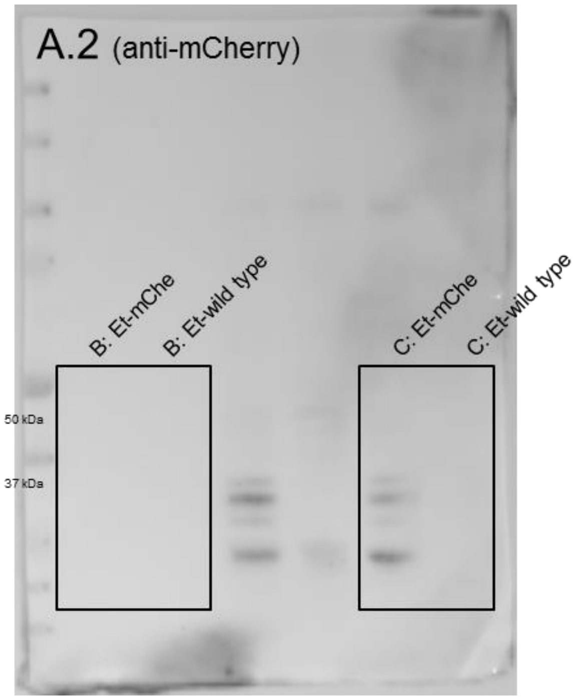
A.2 (anti-mCherry)
B: Et-mChe
B: Et-wild type
C: Et-mChe
C: Et-wild type
50 kDa
37 kDa

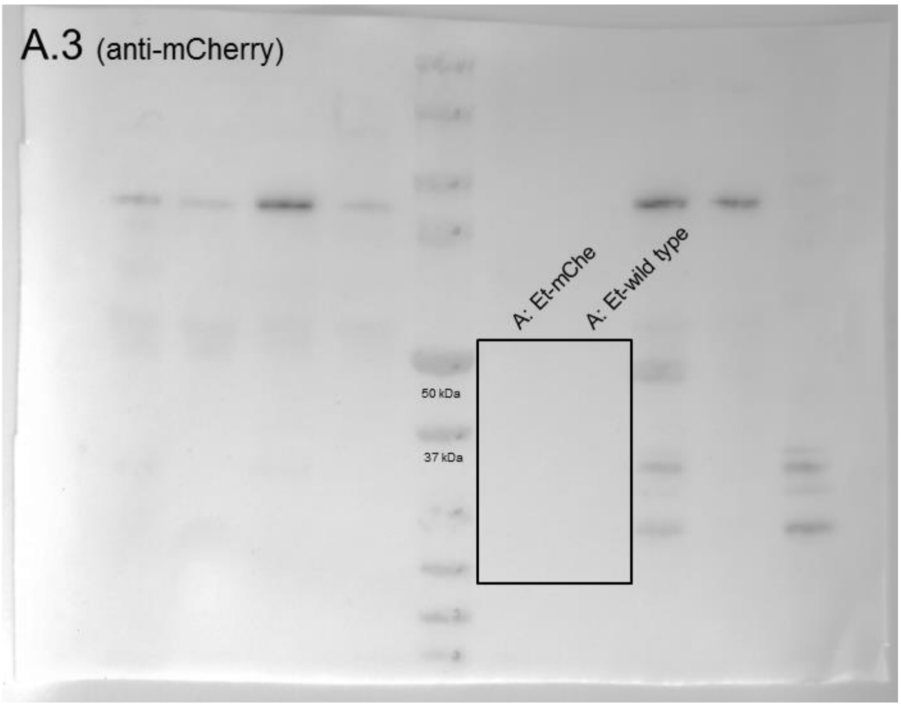
A.3 (anti-mCherry)
A: Et-mChe
A: Et-wild type
50 kDa
37 kDa

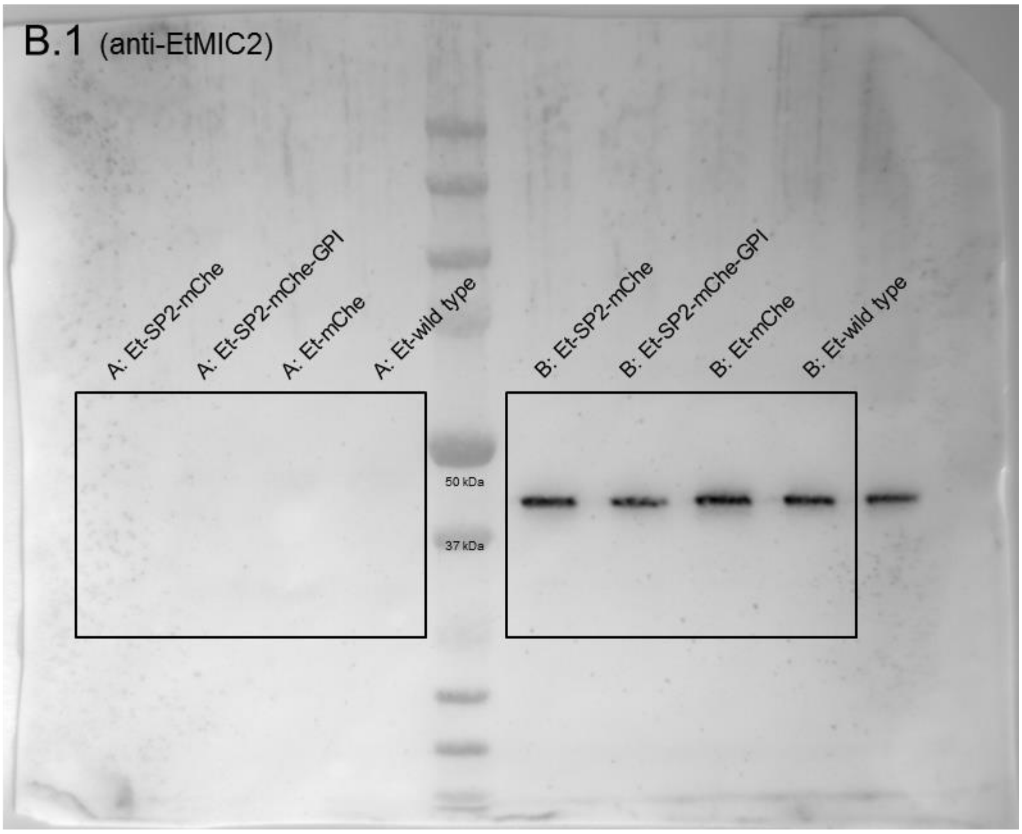
B.1 (anti-EtMIC2)
A: Et-SP2-mChe
A: Et-SP2-mChe-GPI
A: Et-mChe
A: Et-wild type
B: Et-SP2-mChe
B: Et-SP2-mChe-GPI
B: Et-mChe
B: Et-wild type
50 kDa
37 kDa

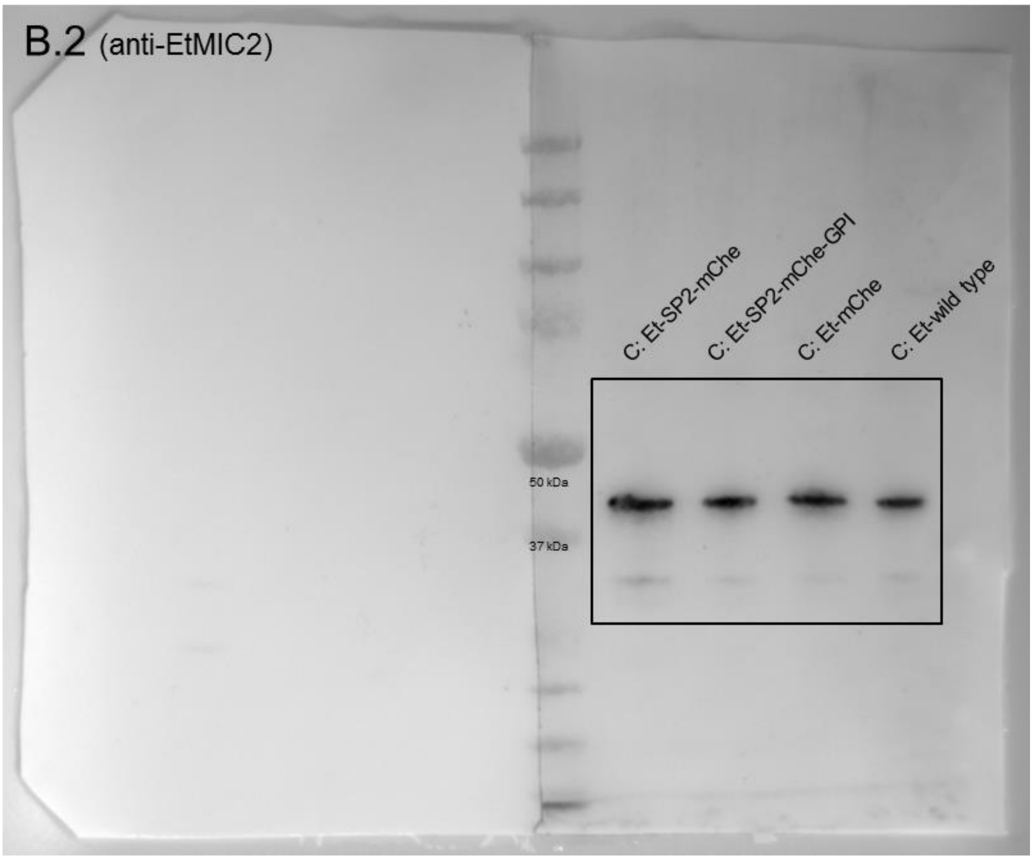
B.2 (anti-EtMIC2)
C: Et-SP2-mChe
C: Et-SP2-mChe-GPI
C: Et-mChe
C: Et-wild type
50 kDa
37 kDa

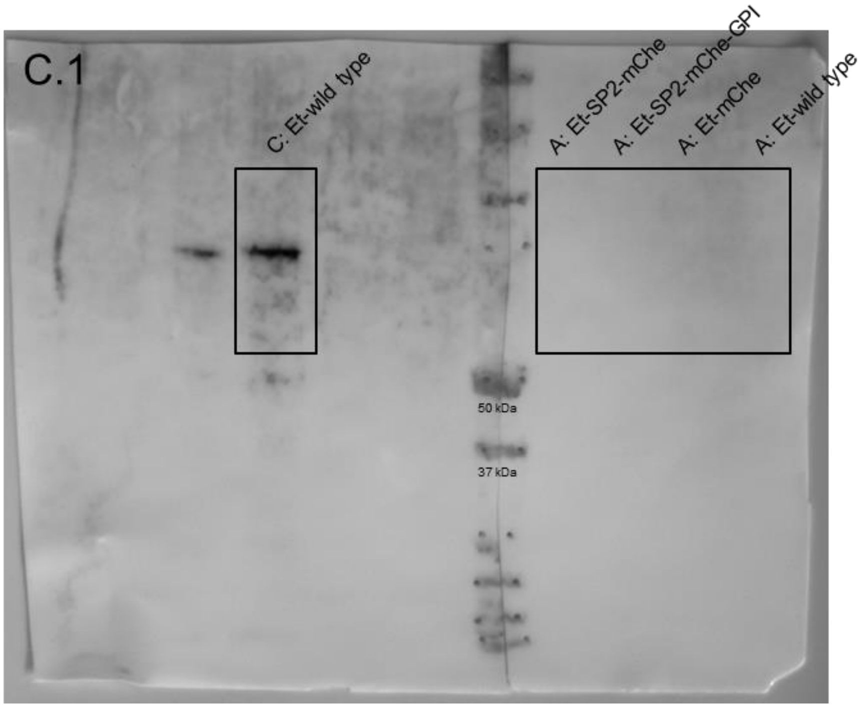
C.1
C: Et-wild type
A: Et-SP2-mChe
A: Et-SP2-mChe-GPI
A: Et-mChe
A: Et-wild type
50 kDa
37 kDa

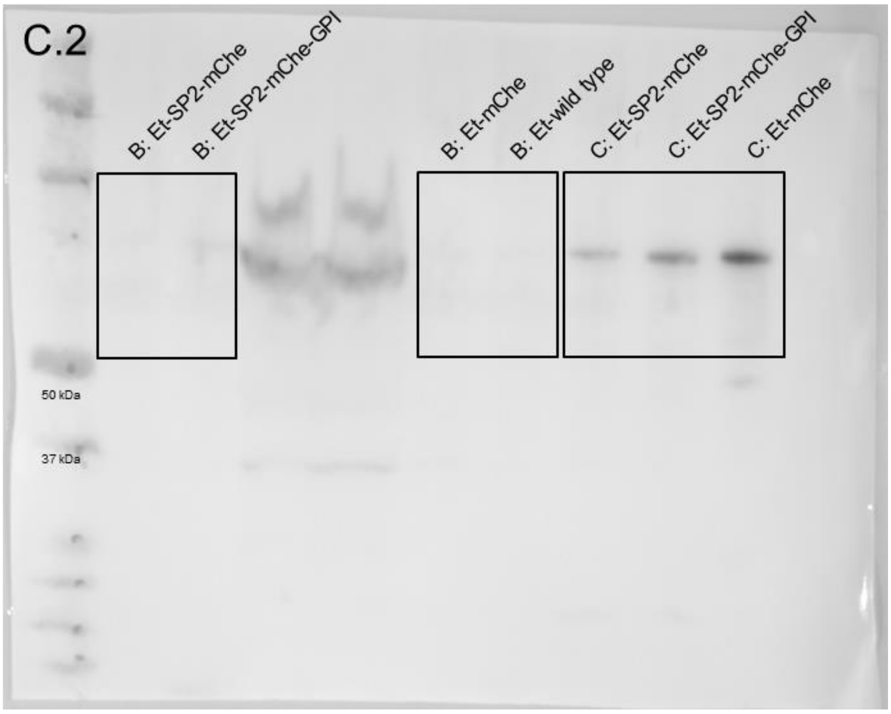

Figure S2. Full length blots from Fig. 3. A (1, 2, 3) anti-mChe antibody as primary antibody. B (1, 2) anti-EtMIC2 antibody as primary antibody. C (1, 2) anti-HSP70 antibody as primary antibody. Corresponding bands are labelled as in Fig. 3.
